# The unlock consequences: changes in daily behaviors and mental health in Indian population during the second wave of COVID-19

**DOI:** 10.5935/1984-0063.20220060

**Published:** 2022

**Authors:** Anshu Dwivedi, Shalie Malik, Sangeeta Rani

**Affiliations:** University of Lucknow, Zoology - Lucknow - Uttar Pradesh - India

**Keywords:** COVID-19, Unlock, Sleep, Eating, Depression

## Abstract

**Objectives:**

The COVID-19 outbreak has led to unprecedented changes throughout the world. It has imposed lockdown, social distancing to avoid the spread of this disease. India in the middle of March 2021 reported the beginning of the second wave of corona leading to massive death. We hypothesized to investigate the effect of sleep and eating behavior also got affected during unlock imposed due to the second wave.

**Material and Methods:**

The data collection was done by using an online google form by making them available to participants through various social media apps via smartphone. Total participants (n=115) mean age was 25.86±9.52 (Mean±SD). The results were analyzed by using RM One-Way ANOVA, Pearson correlation test by using SPSS 26.

**Results:**

We found that the sleep behavior including time to sleep which was delayed by 46min, time to wake up was 58min, and sleep duration was increased by 6min during the unlock days when compared with weekdays before unlock. The eating behavior during unlock including the time of breakfast, lunch, and dinner was delayed by 1-hour 3min, 23min, and 19min, respectively. The social jetlag was reduced by 6min and eating jetlag was increased by approximately 8.4min. We found a strong positive correlation between eating jetlag and social jetlag during unlock (r=0.262, *p*<0.005).

**Conclusion:**

Our findings can help in modifying irregular sleep and unhealthy eating behaviors into a good and healthy lifestyle which will, in turn, lead to a depression-free lifestyle during unlock/lockdown due to the COVID-19 pandemic.

## INTRODUCTION

The COVID-19 has made remarkable changes in human behavior worldwide. It is one of the major threats to humankind. A total of 1.3 billion of the population from different cultures and regions are living in India. So, the government of India has faced a great challenge in controlling the infection of COVID-19 during this pandemic in both first and the second wave. During the first wave of the corona, a nationwide lockdown was strictly imposed on 25^th^ March 2020. This step resulted in the significant reduction of the spread of this virus among the population. The lockdown implementation has resulted in the closure of non-essential activities along with people being forced to live with social distancing confined in their places. Almost after one year of the first wave of COVID-19, the first case was reported on January 2021 in Lakshadweep. By early April 2021, the infection of COVID-19 cases surpassed 1 million active cases in India. At the end of April, India reported 3,500 deaths in one day with the follow-up of 400,000 new active cases^1^. This condition created stress and pressure on the government and frontline workers. The major strain was on the healthcare system including shortage of oxygen cylinders, unavailability of beds, and medicines in the hospitals. This shortage and rapid increase in the death cases per day somewhat impacted our daily lifestyle and mental health too. To tackle the rapid active cases the government of various states implemented night curfew as well as unlock (no nationwide lockdown).

Most of the studies performed during the first wave of COVID-19 addressed that maximum time spent in social media while physical activities and energy levels were reduced^2^, along with the delay in the sleep-wake cycle, sleep duration increased, and poor sleep quality^3^,^4^ and breakfast timings^5^. Also, the population experienced an increase in binge eating which increased the body weight during the pandemic. It has been found that there was a significant association between social and breakfast jetlag before and during the lockdown^3^. Another aspect of COVID-19 has caused a significant negative impact on psychology and mental health in the population^6^. As expected COVID-19 has created an environment of fear and anxiety. Almost 16.5% were severely depressed and 28% were having severe to moderate anxiety symptoms^7^. Studies have also shown that there was a significant increase in usage of social media and its positive association with mental health during lockdown^8^. Home isolation has created a negative effect on psychological and mental health in the population. The individuals were not having any clinical symptoms and were also physically well. Hence, it is necessary to study the effect of unlock on sleep, eating, and mental health of the population.

The objective behind this study is to determine the impact of unlock on our daily behaviors such as sleep including its various parameters like time to sleep, wake up time, sleep duration, social jetlag, and eating habits including time to breakfast, time of lunch, time to dinner, eating duration and eating jetlag. Along with daily behaviors, we also tried to focus on the mental health of the population by knowing their depression levels during the second wave of COVID-19. Secondly, we were interested to know changes adapted during the unlock after getting back to their work from the long gap of lockdown.

## MATERIAL AND METHODS

### Study design and participants

The COVID-19 has led to unprecedented changes in human behavior worldwide. We conducted a cross-sectional study to investigate changes in multiple aspects of sleep and eating behavior during the unlock by comparing before versus during unlock asked in one questionnaire. This study was approved by the institutional ethical committee of the University of Lucknow (LU/IEC/ZOOL/2020/11/06). The data was collected from May 9, 2021 - May 31, 2021, along with a consent form was filled by the participants. Total 115 participants completed the study by giving their correct response out of which 53.91% were female and 46.09% were male.

### Questionnaire

The online distributed questionnaire consists of three sections namely:

**Section 1 -** Demographic information containing name, gender, age, date of birth, and occupation.

**Section 2 -** It contained questions related to sleep and eating variables. The variables of sleep behavior used were sleep onset represented as the time to sleep (TTS), sleep offset as the time to wake up (TTW), sleep duration (SD), mid-sleep duration (MSD). Similarly, variables of eating behavior are the time of breakfast (TOB), time of lunch (TOL), time of dinner (TOD), eating duration (ED), mid-eating duration (MED) before and during unlock. Social jetlag from sleep and eating jetlag from eating behavior was computed, respectively.

**Section 3 -** It contained validated questions of the Centre for Epidemiological Studies Depression Scale (CES-D 8) questionnaire. This questionnaire was used to scale the depression levels in the population. A score of 9 or more is considered clinically significant depression.

### Statistical analysis

The data is analyzed by using RM One-Way ANOVA followed by Bonferroni’s multiple analysis post hoc test. Significance was taken at *p*<0.0001. The Pearson correlation was also used with significance at 0.005. The graph preparation was done by using GraphPad Prism Software version 8.0, San Diego, USA. While for statistical analysis SPSS 26 version was used.

### Assessment of sleep behavior: sleep onset, offset, duration, mid-sleep, and social jetlag

To assess the changes in sleep variables RM One-Way ANOVA was applied followed by Bonferroni’s multiple analysis post hoc test. The participants reported the individual timings of their sleep onset and offset for weekdays, weekends (before unlock), and during unlock. Sleep duration, mid-sleep, and social jetlag were derived^9^,^10^.

### Evaluation of eating behavior: breakfast time, lunch time, dinner time, eating duration, mid-eating duration, and eating jetlag

To evaluate the changes in eating variables participants reported the timings of their breakfast, lunch, dinner before unlock (for weekdays and weekends) and during unlock. Further, eating duration, mid-eating duration, and eating jetlag was calculated^11^. We used RM One-Way ANOVA to determine the significant changes among the different eating behavior before versus during unlock. Further, we applied the Pearson correlation test to determine the relationship between social jetlag, eating jetlag before unlock versus during unlock. The significance was taken at *p*<0.005.

### Estimation of mental behavior: depression during unlock

We have used the Centre for Epidemiological Studies Depression Scale (CES-D 8) to determine the level of depression. The cutoff score of 9 was taken as the significant clinically depressed symptoms. Pearson chi-square was used to see the association of gender and depression.

## RESULTS

**Figure 1:**
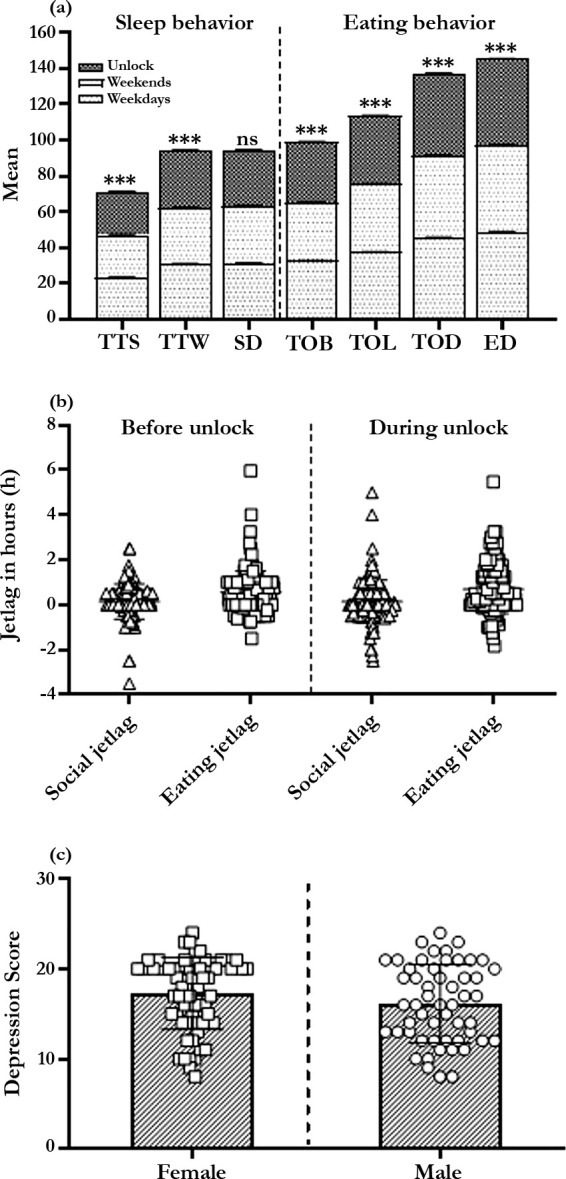
Changes in sleep and eating behavior before (weekdays and weekends) and during unlock. (a) The change in mean sleep and eating behavior before and during unlock. TTS, TTW, SD, TOB, TOL, TOD, ED stands for time to sleep, time to wake up, sleep duration, time of breakfast, time of lunch, time of dinner, and eating duration; (b) The individual plot of social and eating jetlag before unlock days and during unlock days; (c) The individual depression score of female and male during unlock.

We found that about 46.1% were male and 53.9% were female. The total mean age was 25.86±9.52. Out of the total, 53.3% were students, 16.5% were in the private sector, 7.8% were from the government sector, 1.7% had their business, and 15.7% were jobless. The differences in sleep, eating, and depression are shown in the below sections:

**1. Sleep behavior:** the statistical analysis reveals that the sleep parameters significantly changed during unlock as per RM One-Way ANOVA shown in (Table 1). (i) The TTS delayed during unlock by (24.22±0.14, *p*<0.0005) while it was (23.44±0.12, *p*<0.0005) on weekdays before unlock and (23.74±0.13, *p*<0.0005) on weekends. Furthermore, (ii) The TTW increased during unlock (08.27±0.18, *p*<0.0005) while, on weekdays (07.30±0.16, *p*<0.0005) and (07.49±0.16, *p*<0.0005) during weekends before unlock. (iii) Sleep duration of the participants increased by 0.11 h during unlock (7.57±0.12) as it was (7.46±0.13) on weekdays and (7.75±0.10) on weekends reported by the participants (Figure 1a). Overall, the sleep duration was advanced by 0.18h during unlock when compared with weekends before unlock. We also found that there was a strong positive correlation between SD during unlock and SD on weekdays (r=0.431, *p*<0.0001) and with weekends (r=0.50, *p*<0.0001) before unlock.

**Table 1:** Sleep and eating behavior in three different conditions weekdays, weekends, and during unlock days.

		Df	F	*p*	Significance
Time to sleep (TTS)	Weekdays	1.317	34.874	0.0005	^***^
	Weekends				
	Unlock				
Time to wakeup (TTW)	Weekdays	1.869	17.086	0.0005	^***^
	Weekends				
	Unlock				
Sleep duration (SD)	Weekdays	1.979	2.250	0.108	**Ns**
	Weekends				
	Unlock				
Time of breakfast (TOB)	Weekdays	1.902	26.503	0.0005	^***^
	Weekends				
	Unlock				
Time of lunch (TOL)	Weekdays	1.837	11.731	0.0005	^***^
	Weekends				
	Unlock				
Time of dinner (TOD)	Weekdays	1.934	11.510	0.0005	^***^
	Weekends				
	Unlock				
Eating duration (ED)	Weekdays	1.833	11.590	0.0005	^***^
	Weekends				
	Unlock				

Notes: Significance reported from the Repeated-Measure (RM) One-Way ANOVA at *p*<0.0001, Bonferroni post hoc test. Asterisks (*) show significance; Ns shows the non-significance.

**2. Eating behavior:** as per results shown in Table 1. The (i) TOB was (08.74±0.14, *p*<0.0005) on weekdays, (09.51±0.09, *p*<0.0005) on weekends before unlock but it was delayed by (09.80±0.15, *p*<0.0005) during unlock. In the following the (ii) TOL was delayed by (14.10±0.12, *p*<0.0005) during unlock while it was (13.71±0.11, *p*<0.0005) on weekdays and (13.84±0.09) on weekends. The (iii) TOD was delayed by (21.92±0.11, *p*<0.0005) during unlock concerning weekdays (21.59±0.15), while on weekends it was (21.94±0.11). The dinner timings were almost the same on both weekends and during unlock days. (iv) Eating duration during unlock was advanced by 0.73h (12.11±0.17), while it was (12.84±0.15) on weekdays, and (12.43±0.16) on weekends, respectively (Figure 1a and Table 1). The correlation test resulted that there was a strong positive correlation between ED during unlock with ED weekdays (r=0.54, *p*<0.0001) and with weekends (r=0.64, *p*<0.0001) before unlock.

The paired t-test was applied to check whether there was a significant difference in social jetlag during unlock versus before unlock. We found no significant difference in the social jetlag group before and during unlock. Further, the Pearson correlation test was used to determine the relationship between SJL and EJL before versus during unlock (Table 2). The results revealed that SJL before unlock, was 0.15±0.07 and during unlock 0.14±0.03, and EJL before unlock was 0.70±0.10 and during unlock 0.56±0.06, respectively (Figure 1b). The SJL during unlock has a strong positive correlation with SJL before unlock (r=0.420, *p*<0.000). Similarly, EJL during unlock was strongly and positively correlated with EJL before unlock (r=0.56, *p*<0.000). We also found a strong positive correlation between EJL during unlock with SJL during unlock as well as with EJL before unlock (r=0.262, *p*<0.005) (Table 2).

**Table 2:** Relationship between social jetlag and eating jetlag before and during unlock.

		SJLbu	SJLu	EJLbu	EJLu
**SJLbu**	Correlation coefficient	-	0.420^**^	0.266^**^	0.222^*^
	*p*-value		**0.000**	**0.004**	**0.017**
**SJLu**	Correlation coefficient	0.420^**^	-	0.169	0.262^**^
	*p*-value	**0.000**		0.071	**0.005**
**EJLbu**	Correlation coefficient	0.266^**^	0.169	-	0.569^**^
	*p*-value	**0.004**	0.071		**0.000**
**EJLu**	Correlation coefficient	0.222^*^	0.262^**^	0.569^**^	-
	*p*-value	**0.017**	**0.005**	**0.000**	

Notes:

** Correlation is significant at the 0.01 level (2-tailed);

* Correlation is significant at the 0.05 level (2-tailed); SJL = Social jetlag; EJL = Eating jetlag; bu = Before unlock, u = Unlock.

**3. Depression:** we found that about 97.39% of the population was depressed during the unlock. Further, based on gender 98.39% of females and 96.23% of males were depressed. According to Pearson chi-square, there was no statistically significant association between the genders and depression. As per the response, both males and females were equally depressed (Figure 1c).

## DISCUSSION

Due to COVID-19 people were forced to live in social isolation, no work pressure due to closure of schools, colleges, and offices, people tend to shift their sleep and wakeup time towards more delay, late eating habits, no physical activity, spending their maximum time on social media, phone, and television. Apart from delays in their daily behaviors, people experienced a negative impact on their mental health. Likewise unlock has recreated the same situation as it was during the lockdown. Hence, the present study highlights the impact of unlock on daily behavior and mental health in the general population. The results show that different aspects of sleep behavior such as time to sleep, time to wakeup were significantly delayed during the unlock. The sleep time was significantly delayed by 0.78h during unlock. The delay in sleep time during unlock can be due to the increased use of electronic gadgets, social media in the late night^5^.

The participants showed a significant delay in their wake-up time during unlock by 0.97h. The sleep duration was increased by 0.11h. This can be due to a lack of regime, work schedules, closure of schools and workplaces. Due to lack of work schedule, people showed “free-run” which resulted in later sleep time as well as wake up time. This can be supported by our previous study performed during the lockdown, we found a delay in sleep onset and offset time and increased sleep duration in Indian population^5^,^12^,^13^. The reason behind the increase in sleep duration can be that people were able to overcome their sleep debt during the unlock days. As before unlock, people were in the regimented work schedules, regular office, school, and colleges. This work schedule has restricted the amount of sleep, which they tried to overcome during unlock days. Therefore, we can say that unlock during the second wave of corona acted as a lockdown/freedays.

Works of literature have shown that delay in eating habits can disrupt our physiology along with metabolism. One study has reported that misalignment of the eating/fasting cycle and the biological clock has led to a reduction in energy expenditure, leptin, and peptide YY levels^11^. This disruption can result in various health hazards such as obesity^10^, an increase in BMI^11^, etc. According to a study delay in eating time especially breakfast time is more common in the young population^14^. Similarly, our results predict that there was a significant delay in time to breakfast, lunch, and dinner during unlock by 1.06h, 0.39h, and 0.33h, respectively. The reason behind the significant delay in eating behavior during the unlock in our Indian population can be due to late wakeup time^5^. This postponement of eating time can disrupt our natural physiological rhythms leading to various health issues.

A study reported that almost 87% of the working population suffers from social jetlag^15^. In adults, poor health is associated with late sleep timings, social jetlag^16^. In our study we reported a significant positive and strong correlation between social jetlag, eating jetlag computed with during unlock versus before unlock. As we found a significant increase in social and eating jetlag during the unlock, which shows that both are a suitable marker for the misalignment of the internal clock when compared on workdays and freedays. We found a remarkable association between social and eating jetlag, which can be justified by the strong relationship between late sleep and wake up time, resulting in delayed food intake. Hence, we can say that before unlock, because of confined sleep and eating schedule, people were tending to delay their sleep and eating habits during unlock to improve their sleep. Overall, the later sleep, wake up, and eating timings are notable behaviors of the individuals, which are the relevant markers of the disruption of the biological clock leading to various hazardous health issues.

In India, during the second wave of infection massive death occurred which directly impacted the mental health of the population. Our results from the data suggest that about the whole population (97.39%) of the population were depressed during unlock. In our previous study performed during the first wave of COVID-19 about 54% of the population reported depression during lockdown^5^. The depression caused during unlock was increased by 43% in the population. This increase in depression can be due to a sudden rise in death rate caused by the infection of COVID-19, shortage of oxygen, medicines, unavailability of beds in hospitals. The social media, every news channel was having coverage of deaths due to COVID-19 which was one of the major reasons for the depression during unlock. At last, we can say that the second wave of COVID-19 has significantly impacted the daily behaviors and mental health of the general Indian population as it was earlier in the first wave of COVID-19^17^.

## CONCLUSION

Our findings suggest that unlock was almost similar to that of lockdown imposed in the first wave of COVID-19 in India. But the severity of the second wave was more as compared to the first wave, which has significantly affected the mental health of the population. The almost whole population was depressed during unlock. This unlock has resulted in a mismatch of the circadian clock by delaying the sleep-wake cycle, increasing sleep duration during unlock. Hence, due to increase in duration of sleep, individuals tend to have later wake up time which resulted in delayed eating behavior such as time to breakfast, lunch, and dinner. This poor sleep and unhealthy eating behavior can be modified into a good and healthy lifestyle. The government should pay attention to the increase in the cases of mental health and take appropriate action. This study can be further validated by using advanced technologies to determine if this finding applies to the general population.
